# Navigating opportunities and challenges of generative AI in higher education: towards responsible, equitable, and human-centered integration

**DOI:** 10.3389/frai.2026.1750978

**Published:** 2026-03-23

**Authors:** Claudiu Coman, Vasile Gherheș, Anna Bucs, Ecaterina Coman, Cioca Victoria-Rodica, Diana-Cristina Bódi, Raluca-Maria Șerbănescu

**Affiliations:** 1 Faculty of Sociology and Communication, Transylvania University of Brasov, Brașov, Romania; 2 The Academy of Romanian Scientists, Bucharest, Romania; 3 Department of Communication and Foreign Languages, Politehnica University of Timisoara, Timisoara, Romania; 4 Department of Sociology and Social Sciences, University of Craiova, Craiova, Romania; 5 Faculty of Economics and Business Administration, Transylvania University of Brasov, Brașov, Romania; 6 Faculty of Psychology and Educational Sciences, Babeș-Bolyai University, Calea Moților, Cluj-Napoca, Romania

**Keywords:** academic integrity, AI literacy, GenAI, higher education, human-centered AI, policies, regulations, regulatory gaps

## Abstract

The rapid emergence of generative AI (GenAI) tools, such as ChatGPT, presents transformative potential for higher education (HE) while also raising significant ethical, pedagogical, and policy- related concerns. This systematic and thematic review, based on 46 documents from the Web of Science (WoS), narrows its qualitative analysis to 27 peer-reviewed articles to explore key trends in implementation. Findings highlight GenAI’s benefits in improving personalized learning, self-regulated strategies, and adaptive feedback. However, critical issues persist, particularly regarding academic integrity, data privacy, equity, and responsible governance. Core themes include AI literacy, technology acceptance, ethical oversight, and the imperative for culturally responsive integration. The study underscores the need for stakeholder collaboration to ensure the ethical and effective integration of GenAI in education. When applied thoughtfully, GenAI holds significant promise in reshaping educational practices while safeguarding fundamental academic values.

## 1 Introduction

The Fourth Industrial Revolution has made it possible to connect with anyone on the planet and has transformed daily life in countless ways. Recently, the inventions of the Silicon Valley giants have created a world where artificial intelligence (AI) and its tools (ChatGPT, Copilot, Gemini etc.) are increasingly affecting the delicate fabric upon which society functions. In tech companies (such as Microsoft, IBM, and Google), in politics (such as the G7, G20, UN, EU, and EC), and in major institutional bodies (such as the World Bank, World Economic Forum, UNESCO, and OECD), there is growing discussion about the hybridization between what we call human and artificial intelligence. This hybridization, sometimes described as producing a pseudo-human, is viewed by some as the next step in human evolution (Dellermann et al., 2019; Kurzweil, 2005).

Artificial intelligence as a scientific concept was first introduced by Alan Turing in the 1950s, when he described AI as an attempt to replicate the human mind’s ability to learn and make decisions (Haneya et al., 2021). Since then, AI has evolved dramatically. A more recent branch, generative AI (GenAI), consists of algorithms that generate data that mimics existing sources (Chan and Hu, 2023). These algorithms generate translations, explanations, feedback, and ideas, producing outputs that resemble human-authored content based on the prompts we provide (Chang et al., 2023). The development of GenAI has rapidly accelerated in the past decade, reshaping industries, culture, and education.

Among these new technologies, ChatGPT stands out as the most widely used. Since its launch on November 30, 2022, ChatGPT has triggered a wave of publications at a pace that makes a comprehensive review almost impossible, as new contributions appear daily (Bai et al., 2024; Giannakos et al., 2024). Within 2 months of its release, it reached 100 million active users, becoming the fastest-growing application in recorded history (Gherheş, 2018; Sauvola et al., 2024). Its rapid rise illustrates both the promise and the disruption that generative AI brings to society. In higher education, ChatGPT has drawn attention from students, teachers, and administrators alike. It has been explored as a tool for tutoring, personalized feedback, administrative assistance (such as scheduling and guidance), and even for enhancing interactive learning experiences (Mollen, 2025; Bewersdorff et al., 2023; Polonioli et al., 2023).

Exploratory studies show that ChatGPT performs consistently across several tasks. It is effective at creating texts, explaining popular concepts, giving credit when prompted, offering advice, translating, mimicking the user’s writing style, imitating human creativity, and saving time (Bishop, 2023; Gherheș et al., 2025). These capabilities are significant, yet researchers emphasize that the difference between human and AI remains clear: substance, critical thinking, and original ideas are human qualities, while AI replicates style and information without genuine reasoning (Bishop, 2023).

Despite the potential benefits, introducing AI into education comes with risks. Misinformation, plagiarism, academic fraud, polarization, discrimination, and bias are among the issues most frequently raised (Ateeq et al., 2024; Johnson et al., 2024; Pikhart and Al-Obaydi, 2025; Țîru et al., 2025; Miao et al., 2023; UNESCO, 2024). Several AI safety scholars and policy reports argue that advanced AI systems should only be developed when their expected benefits clearly outweigh potential risks and when there is reasonable confidence that key risks can be managed (Chen et al., 2025; He et al., 2025; Bengio et al., 2025). This concern directly applies to universities, which must adapt rapidly to the new technological landscape. Students’ expectations evolve continuously, and higher education institutions must not only address these demands but also ensure that emerging technologies are used responsibly (Gupta et al., 2024; Lia et al., 2024). AI offers tools that can personalize learning, improve assessment, and provide immediate feedback (Sauvola et al., 2024). Yet its responsible use requires the development of ethical competencies among both students and teachers. This includes understanding and respecting privacy regulations, acknowledging original sources, and avoiding misuse of information. For this reason, universities must integrate AI ethics into their curricula through dedicated courses and seminars.

Transparency also plays a critical role. It is essential that academic communities understand how AI algorithm functions, what data sources they use, and what limitations they face. Without such transparency, misinformation and misinterpretation may proliferate, undermining trust in both the technology and in the academic process itself (Xia et al., 2025). When applied thoughtfully, however, AI can not only enhance learning experiences but also bring long-term benefits by optimizing administrative processes and reducing costs (Apolzan and Cîmpineanu, 2024; Wang et al., 2024; Miao et al., 2023). This dual perspective, opportunity and risk, defines the current stage of AI in education.

Despite rapidly growing interest, existing studies often remain fragmented, focusing on technological or pedagogical applications while neglecting regulatory and ethical dimensions. There is limited research addressing how GenAI can be responsibly and inclusively embedded into academic systems (Figueroa De La Fuente and Farhadian, 2025; Garcia-López and Trujillo-Liñán, 2025; Pinho et al., 2025; Bhullar et al., 2024). This paper aims to bridge that gap by systematically reviewing peer-reviewed literature on the integration of generative AI in higher education, with a particular emphasis on responsible governance, equity, and human-centered approaches. The study contributes to current scholarship by synthesizing key debates on the ethical and pedagogical use of GenAI and by identifying practical directions for developing institutional policies that ensure responsible implementation. The following section presents a review of relevant literature to contextualize the research focus.

Building on these considerations, the present study is guided by two research questions that structure the systematic review and thematic synthesis. These questions focus on the factors shaping the adoption of generative AI in education and on the pedagogical frameworks that support its responsible and effective integration.

*Research question 1*: What factors influence the acceptance and equitable implementation of generative AI technologies across diverse educational contexts?

*Research question 2*: How can generative AI be integrated into educational processes to enhance learning outcomes while ensuring ethical compliance and academic integrity?

The two research questions informed the design of the literature search, the selection of studies, and the thematic analysis described in the Materials and Methods section.

### 1.1 Literature review

Globally, education and technology remain closely linked. Technological progress has historically influenced how education is delivered, while educational needs have shaped the development of technology. In the Arab world, a systematic review by Alzahrani, (2022) showed that countries such as Saudi Arabia, the UAE, Libya, Oman, Lebanon, Palestine, and Egypt are beginning to integrate AI into their educational institutions (Haneya et al., 2021). These contexts highlight both opportunities and challenges. Some authors emphasize that AI cannot replace teachers due to structural and cultural barriers (Sourani, 2019). Others report that AI-based environments improve achievement, decision-making, and attitudes toward technology (Abdel Baky, 2022). Additional studies argue that AI offers affordable and personalized education to diverse groups of learners (El-hajj, 2019). Johnson (2022) further shows that AI-related courses generate valuable insights for students and teachers, enhancing adaptation to technological change (Abuzakiyeh, 2018). Institutions themselves play a vital role by facilitating adoption through infrastructure, resources, and awareness programs (Ali and Aysan, 2025).

The concept of human-centered AI (HCAI) captures some of these debates. According to IBM researchers (Geyer et al., 2022), HCAI is an emerging discipline that seeks to create systems that augment and complement human abilities rather than replace them. It emphasizes human control, transparency, fairness, and respect for privacy. In practice, however, HCAI is also linked to the idea of hybridization between humans and machines, which raises deep philosophical and ethical questions. A professor named Dr. Mark Ryan from Wageningen University in the Netherlands has used Michel Foucault’s *The Order of Things* to critique this vision. Foucault (1982) suggested that “man is a recent invention that may disappear, like other historical entities, when something new appears”.

Ryan draws on Foucault’s idea to argue that hybridization poses serious risks for education and society. He identified two main concerns, that defining humanity itself will become increasingly difficult in a hybrid environment, and second, the contradiction of human-centered AI. Individuals may feel pressured to adopt AI, since refusing could leave them disadvantaged compared to others who are faster, more informed, or able to predict events earlier (Ryan, 2024). This dynamic could produce marginalization, discrimination, and polarization within academic and social contexts.

Secondly, Foucault states that the human being in the modern episteme is “a being through whom knowledge will be obtained that will make all knowledge possible” (Foucault, 2001). Ryan’s second concern relates to the flawed reasoning behind HCAI. While its advocates claim that humans remain at the center of development, Ryan argues that in practice, algorithms occupy the true center. This shift in focus highlights the danger of assuming that AI inherently strengthens human agency. Instead, it must be understood as a tool that requires careful regulation, not as an inevitable step in evolution (de Silva et al., 2024; Shneiderman, 2022; Şimşek et al., 2025). The risks of algorithmic profiling illustrate this point clearly. Facebook, for example, reportedly classifies users into 52,000 traits, categories, and subcategories (Alves De Castro and Carthy, 2021). Such classifications reduce human individuality to digital profiles and impose identities constructed by algorithms. Humans remain unique, while AI captures only fragments of behavior through clicks, searches, and online purchases. The result is a digital identity that may distort who we are or who we should be.

These concerns demonstrate the inconsistencies in the human-centered AI framework promoted by international institutions. Within education, adopting such approaches without scrutiny could undermine core values of equity and human development. Instead, universities must proceed cautiously, asking difficult questions and defining universally applicable, safe procedures for integrating AI.

Similar dynamics can be seen in East Asia. In Korea, surveys of 112 students revealed strong interest in AI but low self-assessed knowledge, with significant variation by age (Kim et al., 2022). Only a minority had received formal AI education, underscoring the need for systematic training at both liberal arts and major levels. Similar patterns were observed in a separate exploratory survey conducted among 177 Chinese students in Korea. This survey highlighted that universities increasingly acknowledge the importance of AI for all disciplines and career paths (Lee et al., 2024). Another study found that while students demonstrated average knowledge of AI, they expressed strong confidence in using it for general applications but less so for coding tasks (Chen and Lin, 2024). Overall, they held optimistic views about AI and emphasized the need for courses that explain how it works (Elnagar et al., 2024).

At the same time, limitations are evident. A SWOT analysis of ChatGPT concluded that chatbots can only provide outputs based on their training data, and they are unable to answer complex questions, interpret emotions, or operate reliably without robust infrastructure (Birenbaum, 2023). Birenbaum argues that while ChatGPT may be useful, it must be taught constructively, as it cannot replace human interaction (Birenbaum, 2023). In vocational education, researchers propose new pedagogical models and specialized courses to prepare future teachers to work with AI (Iasechko et al., 2022; Lazorko et al., 2021). Although AI’s role in education continues to expand, integration into university curricula remains limited, requiring further development of educational tools (Iasechko et al., 2022).

Additional research focuses on student satisfaction with ChatGPT. Findings show that perceived ease of use, usefulness, information quality, interaction, and collaborative learning strongly influence adoption, motivation, and satisfaction (Almulla, 2024). Interactive and collaborative strategies encourage engagement and improve how students use ChatGPT for research. At the same time, students express a clear need for accurate, high-quality information (Almulla, 2024). Other studies highlight the importance of balanced human-AI collaboration. In contexts where humans and AI agents interact, systems must be designed to understand human actions and delegate decision-making to the appropriate party. This balance enhances responsiveness, effectiveness, and utility while avoiding overreliance on either humans or algorithms (Fuchs et al., 2022).

Taken together, these findings highlight the dual nature of AI in education. On one hand, generative AI offers tools for personalization, efficiency, and improved learning outcomes. On the other, it raises questions about ethics, equity, and human identity. Recent scholarship emphasizes the need for ethical frameworks and institutional guidance to support the responsible integration of AI in higher education, while maintaining meaningful human oversight in educational processes (Sutedjo et al., 2025). The rapid pace of AI development makes this task urgent.

The literature reveals a growing enthusiasm for integrating AI into higher education, but also exposes significant gaps. Most research emphasizes technical functionality or user perceptions, while fewer studies address governance, ethics, and the long-term societal consequences of AI adoption. There is a pressing need for frameworks that ensure transparency, accountability, and cultural sensitivity in AI integration.

Building on this analysis, the present study systematically examines recent academic contributions to identify emerging patterns, regulatory gaps, and ethical priorities for the responsible implementation of generative AI in higher education (Coelho et al., 2025).

## 2 Materials and methods

This study employed a systematic literature review combined with qualitative thematic synthesis to examine the pedagogical, ethical, and governance dimensions of generative artificial intelligence (GenAI) in higher education. The review followed PRISMA-informed procedures to ensure transparency, replicability, and structured screening.

The analysis was guided by two research questions:

*RQ1*: What factors influence the acceptance and equitable implementation of generative AI technologies across educational contexts?

*RQ2*: How can generative AI be integrated into educational processes while ensuring ethical compliance and academic integrity?

A systematic search was conducted in the Web of Science Core Collection (WoS) database. WoS was selected due to its indexing of high-quality, peer-reviewed journals, its interdisciplinary coverage across education, social sciences, technology, and policy research, and its structured metadata, which supports replicable search procedures and citation traceability. The following Boolean search string was applied: TS = (“generative AI” OR “large language model*” OR “ChatGPT”) AND TS = (education OR “higher education” OR university) AND TS = (regulation OR governance OR policy OR ethics OR “academic integrity”). The search was performed within the Topic (TS) field, which includes Title, Abstract, Author Keywords, and Keywords Plus. This strategy ensured comprehensive retrieval of studies addressing both pedagogical applications and regulatory or ethical considerations of GenAI in higher education. The search was conducted on 15 January 2026 and included all records indexed in Web of Science up to that date.

**Figure 1:**
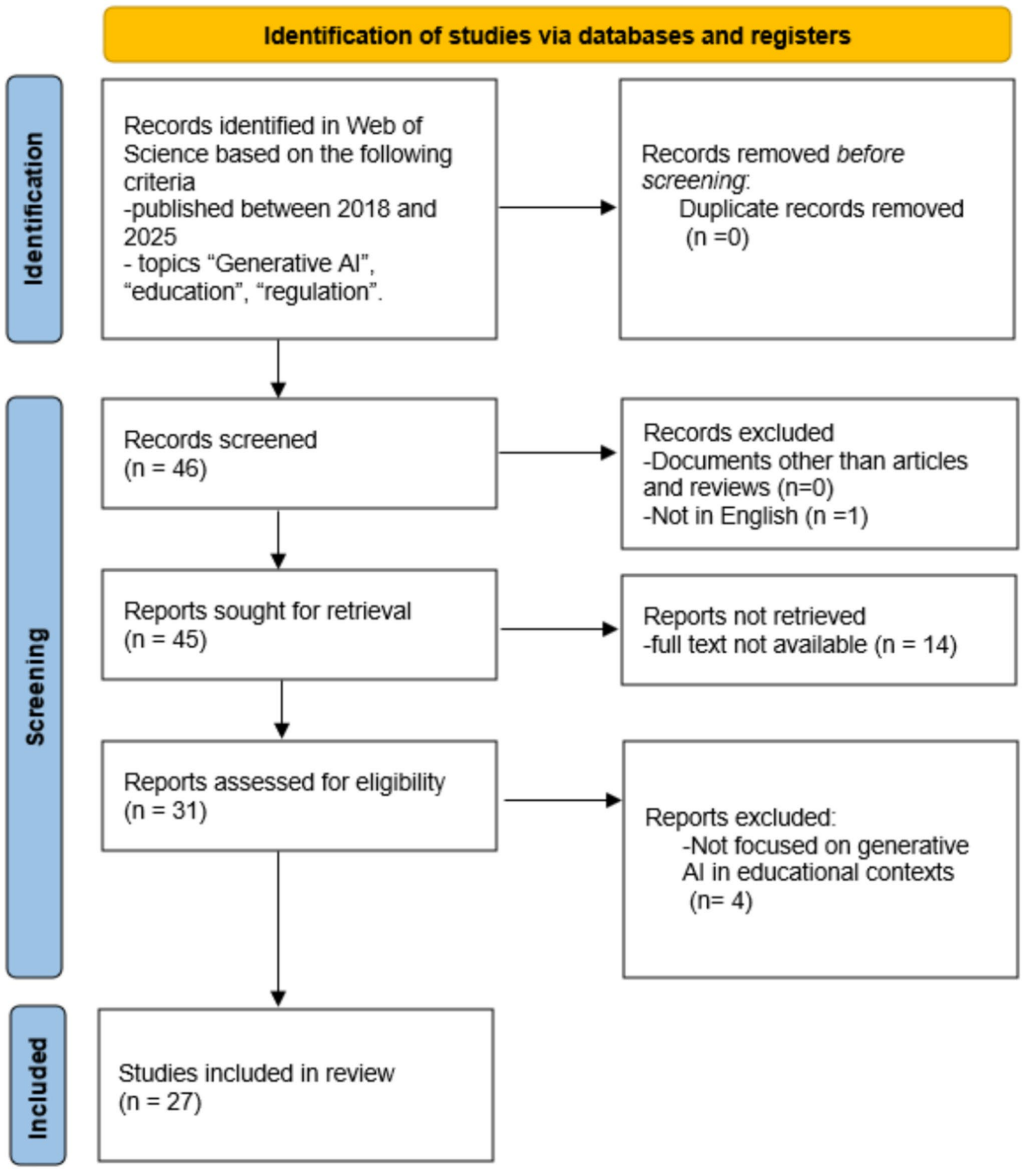
PRISMA workflow.

The initial search returned 46 records, and no duplicates were identified due to the use of a single database. Studies were included if they were peer-reviewed journal articles indexed in WoS, published between 2018 and 2025, written in English, focused on generative AI in educational contexts (particularly higher education), and addressed at least one of the following dimensions: pedagogy, governance, ethics, academic integrity, AI literacy, equity, or institutional readiness. Full-text availability was required to enable in-depth qualitative analysis. Studies were excluded if they focused exclusively on technical model development without educational implications, addressed AI in non-educational sectors, lacked relevance to governance or pedagogical integration, or consisted of editorials or non-peer-reviewed materials. Following title and abstract screening, 19 records were excluded due to limited relevance. After full-text assessment, 27 articles were retained for qualitative thematic synthesis. The study selection process is summarized in the PRISMA workflow (Figure 1).

To enhance completeness, backward and forward citation tracking was conducted for key review articles and high-impact conceptual papers identified during screening. This procedure did not yield additional eligible studies beyond those captured in the primary search. No manual hand-searching of specific journals was performed, as WoS coverage was considered sufficient for the scope of this review.

Bibliographic data were exported into a structured Excel file to support systematic screening and coding. Extracted variables included authorship, publication year, study context, methodological design, thematic focus, and key governance or pedagogical implications. The qualitative synthesis followed Braun and Clarke’s (2006) reflexive thematic analysis framework. The process involved repeated reading for familiarization, open coding of recurring concepts, iterative clustering of codes into provisional themes, and refinement through collaborative discussion among the authors. Initially, ten recurring themes were identified. Through conceptual consolidation and alignment with the research questions, these were synthesized into five higher-order dimensions: pedagogical transformation and learner development; perceptions, acceptance, and responsible use; assessment and academic integrity; ethics, equity, and bias; and governance, institutional readiness, and futures. All authors independently reviewed the coding structure, and discrepancies were resolved through consensus.

This review is limited by the exclusive use of Web of Science and English-language publications, which may exclude relevant regional or non-English studies. Additionally, given the rapid evolution of generative AI technologies, the findings reflect a dynamic and continuously developing research landscape. Despite these constraints, the structured search strategy, transparent eligibility criteria, and systematic thematic synthesis provide a robust analytical foundation for examining responsible and human-centered integration of GenAI in higher education.

## 3 Results

This section reports the results of an inductive thematic synthesis of the Web of Science–indexed studies included in the review. Following reflexive thematic analysis, the analysis initially generated ten recurrent themes, each grounded in multiple empirical or conceptual studies. These themes were identified through open coding and constant comparison, focusing on recurring pedagogical practices, learner and teacher responses, assessment implications, ethical tensions, and governance challenges associated with generative AI in education. In a second analytic step, the ten themes were clustered into five interlocking higher-order dimensions based on conceptual convergence across studies and alignment with established frameworks in learning sciences, AI literacy, ethics, and educational governance (Table 1).

**Table 1:** Five synthesized dimensions and their focus.

Dimension	Focus
1. Pedagogical transformation and learner development	Personalization, SRL and metacognition, mixed-reality/embodied agents, learning-analytics feedback, language learning (Ng and Ho, 2025; Vorobyeva et al., 2025)
2. Perceptions, acceptance, and responsible use	Teacher/student attitudes, AI self-efficacy, TAM determinants, motivation, overreliance and verification habits (Türk et al., 2025; Uludağ et al., 2025)
3. Assessment and academic integrity	Validity under GenAI, process/formative assessment, authenticity, detection/verification, competency-based formats (Zhao et al., 2024; Evangelista, 2025; Kofinas et al., 2025)
4. Ethics, equity, and bias	Data fairness, hallucinations/misinformation, surveillance/privacy, linguistic and socioeconomic disparities (Barus et al., 2025; Garcia-López and Trujillo-Liñán, 2025)
5. Governance, institutional readiness, and futures	Regulatory and institutional frameworks, infrastructure and faculty development, AI literacy, HCAI, participatory governance (Tsao, 2025; Figueroa De La Fuente and Farhadian, 2025; Almatrafi et al., 2024)

### 3.1 Pedagogical transformation and learner development

Across the dataset, GenAI is framed less as a static tool and more as a collaborative partner that actively supports self-regulated learning (SRL) and metacognition. Several studies reconceptualize problem solving as dialogic and iterative, moving through (i) problem understanding, (ii) targeted AI use, and (iii) solution evaluation, explicitly cultivating planning, process, and comprehension monitoring, reflection, and self-explanation. This “pair-thinking” stance positions the chatbot as a competent (but imperfect) peer whose outputs must be interrogated, countering passive consumption and strengthening learner agency (Prasad and Sane, 2024). Designs grounded in Zimmerman’s SRL and Judgments of Learning (JOL) elevate three principles: goal setting (prompting), self-assessment with feedback, and personalization and reposition chatbots as resources of learning rather than answer dispensers; reverse prompts are used to elicit strategy reflection and metacognitive awareness (Zimmerman, 2000; Kong et al., 2024).

When learners attempt to shortcut tasks (e.g., asking for direct answers), AI-supported nudges can redirect them toward deeper engagement, responsibility, and reflective behaviors (Chang et al., 2023). Beyond text interfaces, embodied GenAI agents in mixed reality (MR) increase active learning, shared regulation, and cognitive engagement via multimodal cues while managing cognitive load (Nguyen et al., 2025). The fusion of GenAI with learning analytics (LA) enables real-time, adaptive feedback informed by trace data, but also requires transparency, consent, accountability, and most importantly, ethics by design to maintain learner autonomy and trust (Khosravi et al., 2025).

In language education, systematic and scoping work highlights GenAI’s roles across receptive (reading, listening) and productive (speaking, writing) skills, from primary through adult learning, supporting personalized feedback, SRL, and teacher innovation, while acknowledging technical reliability limits and the risk of over-standardization (Crompton et al., 2024; Kohnke et al., 2025). In broader terms, researchers advocate for a human-centered and glocalized approach to learning design, one that positions large language models (LLMs) as collaborative partners throughout the instructional design cycle, from contextual analysis and idea generation to evidence-based decision making. This perspective acknowledges that, despite their advanced capabilities, deep learning systems ultimately rely on human insight, critical reflection, and authentic problem-solving to achieve meaningful educational outcomes (Giannakos et al., 2024; Fecher et al., 2025). In Vygotskian terms, AI can function as a “more capable peer” in the Zone of Proximal Development (ZPD), offering timely exemplars and feedback that bridge current ability and task demands, provided its use remains strategic and self-regulated (Wang, 2024).

At the mechanism level, multiple studies converge on AI self-efficacy, SRL strategies, cognitive outcomes (e.g., better writing quality, critical thinking, and perceived utility value), underscoring the need to teach the strategic use of AI, not merely to allow it (Jin et al., 2025; Zhou et al., 2024; Ng et al., 2024). Collectively, the literature shows that GenAI’s contribution to cognitive/metacognitive development is substantial when designs foreground learner agency, critical evaluation, and reflective practice.

### 3.2 Perceptions, acceptance, and responsible use

Teacher and student perceptions are best described as cautious optimism coupled with critical awareness. Finnish teacher educators reject simplistic dystopias and see clear value for formative assessment, metacognition, and student well-being, while voicing concerns about trust, learner agency, and divides that could emerge from uneven metacognitive skills; they advocate integrating GenAI as a tool for empowerment rather than surveillance (Vartiainen et al., 2025). Students are widely familiar with ChatGPT, using it for writing and research to save time and simplify workflows, yet they worry about the potential dampening of creativity, originality, and critical thinking. Confusion around institutional rules is common, pointing to the need for clear, consistent policy communication (Krvavica et al., 2025; He et al., 2025).

Acceptance and intention to use generative AI tools are primarily influenced by intrinsic psychological factors. A large scale Technology Acceptance Model (TAM) study based on data from 748 pre-service teachers from 12 universities in Turkey found that metacognitive self-regulation (MSR) and motivation significantly shape perceptions of usefulness and ease of use, which are key predictors of behavioral intention, while subjective norms support initial onboarding but play a smaller role in sustained engagement (Şimşek et al., 2025). Likewise, user experience proves essential. In secondary science education, an SRL-aware generative AI chatbot (SRLbot) enhanced motivation, personalized feedback, and self-regulated learning strategies, including goal setting, environmental structuring, task planning, and time management, outperforming a traditional rule-based chatbot (Ng et al., 2024).

At the same time, overreliance is real: in programming courses, a notable minority delegated entire tasks to ChatGPT without independent problem solving or verification, risking passive learning and reduced self-efficacy: a pattern calling for explicit verification habits and AI literacy (Amoozadeh et al., 2024). Converging evidence indicates that AI self-efficacy predicts SRL, which mediates gains in writing quality and critical thinking, aligning with calls to build confidence, competence, and ethical and reflective use (Jin et al., 2025; Fecher et al., 2025; Elnagar et al., 2024). Institutions can leverage learning analytics to identify usage/engagement patterns and steer personalized supervision, but students’ perceptions and responsible use are undermined when policy clarity and resource access are uneven (Krvavica et al., 2025; Hughes et al., 2025).

In short, both teachers and students recognize potential empowerment, provided that guidelines, training, and metacognitive scaffolds are in place and that overreliance is countered by critical evaluation and verification norms. Despite broad convergence around cautious optimism, important tensions emerge across contexts. For example, while Finnish teacher educators emphasize GenAI’s potential to enhance formative assessment, metacognition, and even student well-being (Vartiainen et al., 2025), Croatian university students report concerns about creativity dampening, originality loss, and over-automation of cognitive effort (Krvavica et al., 2025). This divergence may reflect differences in institutional culture, pedagogical traditions, AI literacy maturity, or policy clarity. Similarly, TAM-based evidence suggests that metacognitive self-regulation and intrinsic motivation drive sustained engagement (Şimşek et al., 2025), yet studies of programming courses reveal a non-trivial subset of students delegating entire tasks to AI systems without verification (Amoozadeh et al., 2024). These findings suggest that self-efficacy and motivation may enable productive use, but without explicit scaffolds for verification and reflective practice, empowerment can quickly shift into dependency.

Moreover, much of the evidence on AI self-efficacy and intention to use relies on large-scale self-report surveys (e.g., Şimşek et al., 2025; Jin et al., 2025), which, while statistically robust, may overestimate responsible usage patterns due to social desirability effects. Experimental or longitudinal designs tracking actual usage behaviors over time remain comparatively limited. As such, current findings on sustained responsible engagement should be interpreted cautiously and warrant further observational validation.

### 3.3 Assessment and academic integrity

GenAI challenges the validity of traditional take-home assessments (e.g., essays, MCQs) by producing high-quality text and answers. A pragmatic response in the literature shifts toward authenticity and process evidence: oral/practical examinations, competency-based evaluations, portfolios, and designs that make metacognition visible (Newton and Jones, 2025; Vartiainen et al., 2025). Finnish educators argue for assessing learning processes (planning, monitoring, reflection) and for formative routines that reduce incentives to cheat by aligning accountability with self-regulatory practice (Vartiainen et al., 2025). Students themselves report ethical dilemmas and uncertainty about expectations, reinforcing the need for transparent policies, AI literacy, and verification protocols (He et al., 2025; Krvavica et al., 2025).

Scholars also reframe integrity through critical literacy with AI as a co-author of meaning, emphasizing responsibility over a narrow focus on “authorship purity” (Kumar et al., 2024). In regulated domains such as health/dental education, the integrity conversation widens to fabricated references, hallucinations, and compliance constraints, mandating rigorous validation, human oversight, and domain specific guidance (Elnagar et al., 2024; Nutbeam and Milat, 2025). Complementing this, technology preparedness tool access, educator training, and policy frameworks help universities adopt assessment formats that preserve validity while enabling constructive AI use (Newton and Jones, 2025; Hughes et al., 2025).

Overall, the trajectory is away from trying to police outputs alone and toward designing for authenticity, competence, and process, supported by policy clarity and ethical pedagogy. Nevertheless, the literature reveals unresolved tensions between process-oriented reform and institutional feasibility. While calls for authenticity and competency-based assessment are conceptually strong (Newton and Jones, 2025; Vartiainen et al., 2025), implementation may be constrained by class size, faculty workload, accreditation requirements, and resource disparities. In highly regulated domains such as healthcare education, where fabricated references and hallucinations carry high stakes (Elnagar et al., 2024; Nutbeam and Milat, 2025), stricter oversight models may be necessary, potentially limiting pedagogical flexibility.

Furthermore, empirical evidence on redesigned assessment effectiveness remains emergent. Many proposals are normative or conceptual rather than empirically validated at scale. Comparative, cross-institutional studies evaluating learning outcomes under redesigned GenAI-integrated assessment models are notably scarce. This represents a critical evidence gap in determining whether authenticity-focused assessment reforms systematically improve learning quality, or whether they merely shift integrity challenges to new formats.

### 3.4 Ethics, equity, and bias

Ethical concerns are both urgent and systemic. A recent thematic analysis foregrounds authenticity, intellectual property, and the opaque provenance of LLM outputs, complicating trust and fairness in educational use (Ali and Aysan, 2025). Finnish teacher educators stress that AI is not neutral, often embedding power asymmetries that can amplify existing inequities; ethical awareness and critical pedagogy are therefore essential (Vartiainen et al., 2025). A posthumanist HCAI lens reframes literacy as co-constructed by humans and technologies, relocating emphasis from proving “human authenticity” toward responsibility in socio-technical meaning-making (Kumar et al., 2024).

The risk of hallucinations and misinformation runs throughout the evidence, prompting calls for routine verification and ethical oversight, particularly in high stakes contexts like healthcare education (Elnagar et al., 2024; Zhou et al., 2024). Surveillance and privacy concerns surface around AI-infused edtech and synthetic media governance; nuanced regulation is needed to protect rights while preserving legitimate creative and educational uses (Broinowski and Martin, 2024). Public and stakeholder studies emphasize manipulation, privacy breaches, misinformation, and unequal access, arguing for proportionate precaution that safeguards fundamental rights without stifling innovation (Machleidt et al., 2024).

Equity concerns are acute in language and access. Training data dominated by English can marginalize less-represented languages/dialects, risk standardization, and disadvantage multilingual learners (Crompton et al., 2024; Ali and Aysan, 2025). Socioeconomic disparities bandwidth, device quality, subscription costs create nontrivial barriers and uneven policy awareness, undermining responsible adoption (Krvavica et al., 2025; Hughes et al., 2025). While LA + GenAIcan personalize support and identify learners at risk, poorly governed systems may reproduce bias; privacy-preserving, transparent designs that uphold learner data rights are therefore non-negotiable (Khosravi et al., 2025). In specialized fields, gaps in domain coverage and regional relevance further complicate equitable outcomes, another reason to invest in inclusive datasets and context-sensitive AI literacy (Elnagar et al., 2024; Newton and Jones, 2025).

Taken together, the ethical-equity literature insists on fairness by design, verification literacy, and rights-preserving oversight as the conditions under which GenAI can be responsibly mainstreamed. At the same time, tensions persist between precautionary regulation and innovation enablement. While several scholars advocate proportionate precaution to mitigate risks of misinformation, privacy breaches, and algorithmic bias (Machleidt et al., 2024; Broinowski and Martin, 2024), others caution that overly restrictive governance could hinder pedagogical experimentation and equitable access to AI-supported learning opportunities. This regulatory ambivalence is especially pronounced in multilingual and lower-resource contexts, where the absence of locally adapted datasets may simultaneously justify caution and necessitate accelerated innovation (Crompton et al., 2024; Ali and Aysan, 2025).

Methodologically, much of the current evidence on bias and hallucination risk is based on thematic analyses, stakeholder perceptions, or domain-specific case examples rather than large-scale empirical auditing of model outputs across diverse educational settings. Systematic bias audits and multilingual benchmarking studies remain underdeveloped. As such, while ethical risks are convincingly theorized, empirical quantification across disciplines and demographic groups remains limited.

### 3.5 Governance, institutional readiness, and futures

Policy and institutional responses have lagged GenAI’s rapid evolution. In Europe, risk-based regulatory trajectories (e.g., the AI Act’s approach to general-purpose AI) offer scaffolding, yet enforcement and liability remain complex for fast-changing LLMs and education use cases. Scholars caution that prevailing framings are often STEM-centric, underweighting cultural/creative considerations and educational values; they argue for human-centered, context-sensitive, and participatory governance with continuous monitoring (Ali and Aysan, 2025; Machleidt et al., 2024; Broinowski and Martin, 2024).

At the institutional level, readiness spans (i) infrastructure and access (including licensing), (ii) faculty development, (iii) clear policies and assessment redesign, and (iv) equitable resourcing. Without these, adoption risks widening digital divides and intensifying workload pressures (Hughes et al., 2025; Newton and Jones, 2025). Finland’s emphasis on inquiry and formative assessment provides a conducive base for responsible integration, while collaborative design among educators, instructional designers, and developers aligns tools with SRL and personalization goals (Vartiainen et al., 2025; Chang et al., 2023; Khosravi et al., 2025). Crucially, students’ primary stakeholders should co-create AI policies and assessment norms; policing-only approaches are insufficient compared to dialogue about purpose, learning value, and shared standards (Bai et al., 2024).

Looking forward, the field envisions human centered AI (HCAI) futures where agency and oversight remain central while GenAI augments personalized, sustainable learning at scale. Realizing this vision requires intentional pedagogy, ethics-by-design, inclusive datasets, and institutional clarity, and in regulated domains, unwavering attention to compliance, validation, and safety (Giannakos et al., 2024; Fecher et al., 2025). Cross-context comparison further indicates that institutional readiness varies significantly by national policy culture, disciplinary tradition, and infrastructural capacity. Finland’s inquiry-based pedagogical culture appears conducive to SRL-aligned integration (Vartiainen et al., 2025), whereas institutions reporting policy ambiguity and uneven resource distribution demonstrate higher levels of student uncertainty and inconsistent usage norms (Krvavica et al., 2025; Hughes et al., 2025). These contextual differences suggest that GenAI adoption is not merely technological but deeply institutional and cultural.

Importantly, longitudinal research on sustained GenAI integration beyond initial adoption phases remains largely absent. Most studies capture early implementation or pilot-stage interventions. Long-term trajectories—particularly regarding faculty workload, curriculum redesign sustainability, and student skill transfer beyond course boundaries—remain empirically underexplored. Future research should therefore prioritize multi-year, cross-institutional studies that track both pedagogical outcomes and governance adaptation over time.

## 4 Recommendations

### 4.1 Recommendations for policymakers

We urge policymakers to create regulatory systems that are flexible, corresponding to rapidly evolving AI. These systems should not only be co-created with educators, technologists, and ethicists but also include students and community representatives to guarantee that they are context-sensitive and inclusive. Regulations should clearly specify data privacy, algorithmic transparency, intellectual property rights, and provisions for accountability. In particular, regulations could require AI systems to be audited for bias and fairness, reveal the use of AI in education, and implement ethical AI usage standards in sensitive sectors like healthcare and education (Mittelstadt, 2025).

To avoid situations where educational opportunity gaps exist, governments and educational authorities should develop a digital infrastructure to ensure that all learners have equitable access to AI tools. The infrastructure can provide the service of accessing AI tools either at a low price or free of charge for those who cannot afford it. Moreover, as the infrastructure is being built, AI literacy programs should be developed and introduced into the education system at all levels, with no exceptions. These programs should focus on the ethical use of AI, critical thinking, and self-regulation skills. The programs should be culturally and linguistically inclusive. They should be tailored for different communities in order to bridge the digital divide and support the most disadvantaged ones. For example, the funding may be used for local AI models that are trained by giving them the datasets that represent the diversity of the languages and contexts. As Selwyn (2024) highlights, inequality in access to digital resources directly correlates with disparities in learning outcomes, and without infrastructure investment, AI can intensify these gaps rather than ameliorate them.

Morally principled decision makers have the responsibility to demand that schools are clear about their AI usage policies and that they also set clear guidelines for students on academic integrity, as well as the permissible work that is AI-assisted and the consequences of misuse. Schools should be obliged to inform the corresponding oversight bodies about the achievements and problems concerning AI deployment at regular intervals. Moreover, these rules ought to specify the application of student-friendly analytics that comply with student privacy regulations and empower students with the right to decide what happens with their data, which in turn will build confidence and encourage responsible participation in the AI field.t w.

### 4.2 Recommendations for teachers

Teachers must make AI literacy a definite part of their teaching, giving students the skills to understand how AI systems operate, know their weaknesses, and have a sense of ethics. This part includes training students to be able to critically assess AI-generated content, detect possible biases or hallucinations, and adopt AI to help in a responsible way in their learning processes. Arts-based classroom activities could consist of short texts where students observe, compare AI outputs with those generated by peers, journals of their reflections on AI use, and debates on AI’s impact on society (Anders and Speltz, 2025).

Evidence consistently shows that metacognitive self-regulation (MSR), intrinsic motivation, and AI self-efficacy significantly predict sustained and meaningful GenAI adoption (Şimşek et al., 2025; Jin et al., 2025; Zhou et al., 2024), while SRL-aware implementations enhance goal setting, planning, and time management (Ng et al., 2024). At the same time, traditional assessment formats are increasingly vulnerable to AI substitution (Newton and Jones, 2025), whereas process-oriented and competency-based models better align with self-regulated learning and reduce integrity risks, particularly in domains sensitive to hallucinations or fabricated references (Elnagar et al., 2024; Nutbeam and Milat, 2025; Vartiainen et al., 2025). Accordingly, GenAI integration should explicitly scaffold planning, monitoring, reflection, and verification routines, positioning AI as a strategic learning partner, while assessment should prioritize authentic, process-visible formats (e.g., oral defenses, portfolios, staged drafts, reflective commentaries) over outcome-only evaluation.

Teachers should rely on AI as a partner when working within SRL frameworks, thus they encourage learners to involve themselves in metacognitive activities, by setting goals, planning, controlling, and reviewing the use of AI tools. For instance, teachers can come up with assignments that allow students to interact with AI repetitively by changing prompts, judging if the solution is correct, and modifying their answers to this, which is the case of deep cognitive involvement instead of passive reception. Accountability and critical thinking can also be enhanced by incorporating peer review and portfolio tracking phases (Anders and Speltz, 2025).

Due to AI’s capability to come up with academic work of very high quality, it has become necessary for educators to rethink their assessment strategies if they want to be able to genuinely find out what students have learned. Such methods are prioritizing oral exams, practical demonstrations, project based evaluations, and real-time problem-solving exercises, which are all geared towards students showing their understanding of AI-generated content beyond mere reproduction. Additionally, the use of AI in assessments that are open and transparent, such as when AI is employed to create sample questions or generate individualized feedback, can be a way to benefit from AI while maintaining academic integrity (Kickbusch et al., 2025).

When AI tools are introduced in the classrooms, teachers need to be extremely careful not to take away the students’ linguistic and cultural diversity. That means the positive action of selecting AI programs that are capable of giving multi-lingual and multi-dialectal support, recognizing the possible prejudices that can arise in AI output, and finding ways to resolve them. To achieve this, teachers can lead discussions on which standard languages and cultures would be most properly represented in AI, while inspiring students to think critically about their engagement with AI. This approach gives them the space to voice their identities and become active participants in the equity struggle (Tao et al., 2024).

Teachers have to acknowledge that literacy is not just a human-centred concept, but it is the result of interactions between humans, technologies (for example, LLMs), environments, and histories. Additionally, creating meaning is just as physical as it is symbolic or linguistic. Teachers are advised to transition from:

From intentionality to responsibility (or response-ability): They need to emphasize that the idea of using AI in writing is not about stealing or being lazy, but rather about how agency and literacy are distributed and performed throughout complex systems.

From authenticity to mattering: Rather than wondering if the output is “authentically human,” teachers should be concerned with what is essential, whose voices get to be heard, and who is left out of the discussion.

From imitation to multifarious communication: The language of AI-generated content is not merely copying; it is profoundly changing the very nature of expression and communication over different media, languages, and cultures.

AI not only enables writing by automation; it is the very mechanism through which literacy is performed, and the concept of being literate in the 21st century is redefined. This view aligns with critical literacy and new literacy studies and even extends beyond questioning the human dominance in the process of meaning-making. We invite teachers to critically evaluate AI tools with students, and thus, the students will become aware of:

- The concealed work, environmental influence, and prejudices are deeply rooted in the AI systems.
- The ways of utilizing generative AI for becoming more creative and for expressing in a responsible manner.
- The fact that digital literacy should involve the analysis of whose knowledge and whose speaking or writing practices are made normal and whose are marginalized or silenced.

## 5 Discussion

We conducted a systematic review with thematic synthesis that investigated the use of generative AI in education. We particularly examined the trends of pedagogical innovation, ethical issues, acceptance of technology, intellectual growth, equality, and regulation in education. Our results enabled us to understand in depth how AI instruments, such as ChatGPT, are changing the teaching and learning process, and at the same time, they help us identify the current challenges.

Our thematic analysis of recent publications and empirical studies revealed that GenAI is a powerful resource for personalizing learning, facilitating self-regulated learning (SRL), and providing adaptive feedback to students. The “pair thinking” dialogic model involves students who actively participate and use AI as a co-working partner, thus allowing them to develop their metacognitive skills, such as planning, reflecting, and critical evaluation. It is a direct way of addressing the concerns raised about the passive nature of consuming AI-generated materials, and at the same time, it enables learners to become agents of their own learning (RQ1). Pedagogical frameworks based on Zimmerman’s SRL model (Kong et al., 2024) tend to emphasize goal-setting, self-assessment, and personalized feedback as the primary requisites for responsible AI use. On the other hand, ethical problems, which are represented by consequences of misinformation, academic integrity, domain-specific limitations, and so on, imply a need for strong institutional policies and continuous human oversight to ensure that AI does not become the cause of the problem but rather a solution to education quality.

The findings clearly show that intrinsic cognitive factors, among which metacognitive self-regulation and learning motivation are strong indicators of educators’ and students’ acceptance of AI tools. This relationship is supported by recent evidence showing that AI literacy and 21st-century skills significantly predict students’ intention to adopt generative AI in academic contexts (Salhab and Aboushi, 2025). The social impact, although it plays a role at the beginning of the adoption process, is not as strong during the actual use. Besides, infrastructural readiness, clear policies, and equitable access thus emerge as the most important systemic enablers. The digital divide and linguistic biases in AI models are sources of risk in exacerbating existing educational inequities. Hence, the culturally responsive AI designs and inclusive governance frameworks become indispensable if fair access and representation are to be guaranteed. Recent evidence confirms these concerns, showing that many AI systems used in higher education continue to reproduce linguistic and cultural inequities when training data are not representative (Conceição and van der Stappen, 2025). Learning analytics combined with GenAI may offer the students personal insights and assistance, yet privacy protection and learner empowerment must be priorities in an ethical design of such a system (RQ2).

The results of our study are in accordance with the general opinion that AI in education is not the magic solution, nor the only problem; if taken alone, its influence is conditional upon intentional, context-sensitive implementation. The idea of hu-man-centered AI (HCAI) provides an opportunity for educators and policymakers to keep human agency as well as critical oversight while they decide which parts of AI will be most helpful, without falling into the trap of techno-deterministic narratives that either exaggerate or deny the role of AI. Ethical governance has to be flexible, adapting to the changes by involving multi-stakeholder dialogue and continuous monitoring in order to be able to respond to new situations such as surveillance, misinformation, and bias. Recent work supports this view, showing that trust and acceptance of educational AI differ significantly across students, teachers, and parents, and that transparency and clear human oversight are essential for responsible adoption (Karran et al., 2025).

The findings further underscore the need for the overhaul of assessment paradigms towards realistic, competency-based evaluations rather than mere AI-assisted outputs. These include, for example, oral exams, practical assessments, and portfolio-based evaluations, which are underpinned by clear institutional guidelines to ensure that academic integrity is not compromised. This perspective is supported by recent findings showing that traditional essay-based and multiple-choice assessments lose validity when generative AI can produce high-quality responses with minimal human input (Newton and Jones, 2025).

Across the five synthesized dimensions, a set of recurring tensions emerges that define the current phase of generative AI integration in education. First, there is a tension between empowerment and dependency. While GenAI demonstrably enhances metacognitive scaffolding and self-regulated learning when strategically deployed (Ng et al., 2024; Jin et al., 2025), evidence also shows patterns of overreliance and task delegation (Amoozadeh et al., 2024). This suggests that GenAI does not inherently strengthen or weaken learning; rather, outcomes depend on whether instructional design foregrounds regulation and verification norms.

Second, a tension exists between innovation and integrity. Authentic, process-based assessment models are increasingly advocated (Newton and Jones, 2025; Vartiainen et al., 2025), yet institutional feasibility and regulatory constraints complicate rapid redesign, particularly in high-stakes professional fields (Elnagar et al., 2024). Thus, integrity reform must be pedagogically ambitious but structurally realistic.

Third, there is a structural tension between personalization and equity. While GenAI and learning analytics enable adaptive feedback at scale (Khosravi et al., 2025), disparities in access, linguistic representation, and policy clarity risk reproducing or amplifying inequality (Crompton et al., 2024; Hughes et al., 2025). Responsible integration therefore requires equity-by-design rather than retroactive mitigation.

Fourth, governance faces a tension between precaution and participation. Regulatory approaches emphasizing risk mitigation (Machleidt et al., 2024; Broinowski and Martin, 2024) must be balanced with participatory, human-centered AI frameworks (Kumar et al., 2024; Giannakos et al., 2024) that preserve educational innovation and agency.

Taken together, these tensions suggest that the central question is not whether GenAI should be adopted, but under what pedagogical, ethical, and institutional conditions it enhances rather than displaces human learning processes. The review therefore contributes a conditional integration model: GenAI is educationally beneficial when embedded within metacognitive scaffolding, authentic assessment, equity safeguards, and participatory governance; absent these, risks of dependency, inequity, and erosion of integrity increase.

## 6 Conclusion

Generative AI has the potential to make a positive impact on education by creating personalized, adaptive, and engaging learning environments. If AI is put to use intelligently, it can help cognitive and metacognitive development, enable learner autonomy, and improve pedagogical practices. Nonetheless, these advantages will only be realized if we address ethical, equity, and regulatory issues proactively.

The research we conducted highlights that interdisciplinary cooperation among educators, technologists, policymakers, and learners is essential if we want to develop AI models that are suitable for local contexts, ethical frameworks, and inclusive policies. Priority should be given to ensuring equitable access and cultural responsiveness to prevent the exacerbation of educational disparities. Transparent communication about the powers of AI as well as its limitations is a prerequisite for building trust and accountability.

In conclusion, the prospects of AI in education depend on the representativeness of ethical and cultural decisions, which are informed and take into account the responsibilities that accompany innovations. By recognizing AI as a partner rather than a substitute, education has the potential to become a more personalized, diversified, and efficient process.

## 7 Study limitations

We acknowledge several limitations to the study. Most of the literature and datasets used primarily focused on higher education institutions located in a few regions of the world, such as Turkey, Finland, Hong Kong, and Croatia. The situation may differ worldwide because educational policies, technology infrastructure, and people’s attitudes towards AI in general can vary significantly. Therefore, if a conclusion is drawn only on such limited data, it cannot be declared as a universal truth for all countries, especially for those that are economically disadvantaged. Future research should focus on a more varied educational setting and learner demographics to discuss the issue in a more global context.

AI tools like ChatGPT, among other generative AI technologies, are growing at an extremely fast rate. The descriptions, limits, and implications for society of these instruments are in a state of constant flux, making it difficult for researchers relying on available data and literature to capture the complete picture. Thus, some findings may not align with future reality as AI developments continue or the regulatory environment evolves. Research over longer periods and continual observation can help document the constantly changing situation of AI in education.

Much of the recent research, including the one analyzed in this study, focuses on the short period immediately following the implementation of AI in schools, where changes in students’ motivation, learning engagement, and assessment integrity are the primary topics. We observe a lack of empirical studies on long-term educational outcomes, such as continuous cognitive improvement, development of critical thinking skills, and equity issues.

The ethical, legal, and policy aspects of artificial intelligence are complex. They exist alongside rapidly changing regulations in various parts of the world. Our review pointed out these difficulties and is still limited by the different management types and unstable nature of these governance models. Such a situation makes it challenging to develop universally acceptable solutions due to the absence of a standardized ethical code and the existence of inconsistent regulations.

Generative AI includes a broad spectrum of technologies, including images, audio, and virtual beings; however, this paper primarily focuses on text-based AI tools, such as ChatGPT. Now, these tools are most commonly adopted in educational settings, but still, in the future, studies will have to cover more different AI forms in order to be comprehensive in understanding the impact of AI on education.

## Data Availability

The original contributions presented in the study are included in the article/Supplementary material, further inquiries can be directed to the corresponding author.
