# Supplementary material for: Navigating opportunities and challenges of generative AI in higher education: towards responsible, equitable, and human-centered integration
Source: Front Artif Intell. 2026 Mar 23;9:1750978. doi: 10.3389/frai.2026.1750978 (PMC13050895; doi:10.3389/frai.2026.1750978)
Supplement: Supplementary file 1 [file Table_1.docx]

APPENDIX A

| **Full title** | **Authors** | **Methods (from accessible abstract/text)** | **Key results (from accessible abstract/text)** |
| --- | --- | --- | --- |
| *A Self-Regulated Learning Framework Using Generative AI and Its Application in CS Educational Intervention Design* | Prasad, P.; Sane, A. | Conceptual paper proposing a self-regulated learning (SRL) framework for novice programmers using generative AI; focuses on intervention design rather than empirical testing. | Conceptualizes GenAI as interacting with learners’ self-regulation processes; outlines an SRL-guided design space for educational interventions that emphasize planning, monitoring, and reflection rather than answer provision. |
| *Attaining Self-Regulation: A Social Cognitive Perspective* | Zimmerman, B. J. | Theoretical chapter (non-empirical) presenting a social-cognitive model of self-regulated learning. | Provides the foundational SRL model (goal setting, self-monitoring, self-evaluation) widely used to frame learning designs and metacognitive scaffolding. |
| *A Pedagogical Design for Self-Regulated Learning in Academic Writing Using Text-Based Generative AI Tools: 6-P Pedagogy* | Kong, S.-C.; Lee, J. C.-K.; Tsang, O. | Conceptual pedagogical design paper proposing a six-phase framework (Plan–Prompt–Preview–Produce–Peer-review–Portfolio-tracking); explicitly notes lack of empirical validation. | Argues that structured phases, verification, and reflective portfolio work can foster SRL, critical thinking, and academic integrity when using GenAI for writing. |
| *Educational Design Principles of Using AI Chatbot That Supports Self-Regulated Learning* | Chang, D. H.; Lin, M. P.-C.; Hajian, S.; Wang, Q. Q. | Conceptual design-principles paper grounded in SRL and Judgments of Learning theory; no original empirical study reported. | Proposes that chatbots should scaffold goal setting, feedback-based self-assessment, and personalization, positioning AI as a learning resource rather than an answer generator. |
| *Re-Examining the Future Prospects of Artificial Intelligence in Education in Light of the GDPR and ChatGPT* | Bai, J. Y.; Zawacki-Richter, O.; Muskens, W. | Conceptual and governance-oriented analysis revisiting earlier AI-in-education scenarios in light of GDPR and ChatGPT. | Highlights regulatory, assessment, and governance implications of generative AI; emphasizes participatory approaches and student agency in institutional AI policies. |
| *Designing Embodied Generative Artificial Intelligence in Mixed Reality for Active Learning in Higher Education* | Nguyen, A.; Gul, F.; Dang, B.; Huynh, L.; Tuunanen, T. | Design Science Research study developing an embodied GenAI system in mixed reality; evaluated with 26 higher-education learners. | Reports increased learner engagement and active learning; examines how embodied GenAI can support learning while managing cognitive load in immersive environments. |
| *Generative AI and Learning Analytics: Pushing Boundaries, Preserving Principles* | Khosravi, H.; Shibani, A.; Jovanovic, J.; Pardos, Z. A.; Yan, L. | Editorial and conceptual synthesis framing a special issue; uses learning analytics theory (e.g., Clow’s cycle) to organize emerging work. | Positions learning analytics as a lens to evaluate GenAI’s impact on learning and agency; argues for human-centered, transparent, and privacy-preserving integration of LA and GenAI. |
| *AI and English Language Teaching: Affordances and Challenges* | Crompton, H.; Edmett, A.; Ichaporia, N.; Burke, D. | Systematic review (PRISMA-guided) synthesizing 42 studies on AI in English language teaching (based on abstract). | Identifies affordances such as personalization and feedback alongside challenges including reliability, bias, and over-standardization; calls for nuanced and context-aware adoption. |
| *Microlearning and Generative AI for Pre-Service Teacher Education* | Kohnke, L.; Zou, D.; Xie, H. | Qualitative case study over 13 weeks with 19 pre-service teachers using microlearning modules with GenAI. | Reports progressive development of teachers’ AI integration capability, emphasizing hands-on microlearning to support TPACK and SRL. |
| *Students’ Voices on Generative AI: Perceptions, Benefits, and Challenges in Higher Education* | Chan, C. K. Y.; Hu, W. | Empirical survey study (Likert-scale and open-ended responses) with university students in Hong Kong. | Students express generally positive views on GenAI’s usefulness but report concerns about ethics, plagiarism, creativity, and academic integrity. |
| *ChatGPT and Imaginaries of the Future of Education: Insights of Finnish Teacher Educators* | Vartiainen, H.; Valtonen, T.; Kahila, J.; Tedre, M. | Workshop-based qualitative exploration with teacher educators (abstract-level description). | Educators recognize pedagogical potential but stress ethical concerns, learner agency, trust, and the risk of widening divides; advocate responsible integration. |
| *Students’ Attitudes Towards the Opportunities and Challenges of Generative Artificial Intelligence in the Academic Environment* | Krvavica, A.; Papić, A.; Mićunović, M. | Empirical questionnaire-based survey study (full PDF accessible). | Students widely use GenAI and acknowledge benefits but emphasize the need for clear ethical guidance, policy clarity, and safeguards against misuse and overreliance. |
| *AI-Generated Content and Academic Norms: Perspectives and Reflections of College Students* | He, Q.; You, X.; Shen, J. | Empirical study based on student perspectives (abstract-level information). | Highlights ethical dilemmas, uncertainty about academic norms, and tensions between efficiency and integrity under GenAI use. |
| *Extending the TAM Framework: Exploring Learning Motivation and Agility in Educational Adoption of Generative AI* | Şimşek, A. S.; Cengiz, G. Ş. T.; Bal, M. | Large-scale quantitative study (n = 748 pre-service teachers) using TAM extension and structural modeling. | Finds that metacognitive self-regulation and motivation shape perceived usefulness and ease of use; subjective norms matter mainly for initial adoption. |
| *Empowering Student Self-Regulated Learning and Science Education through ChatGPT* | Ng, D. T. K.; Tan, C. W.; Leung, J. K. L. | Comparative empirical study comparing an SRL-aware GenAI chatbot with a rule-based chatbot in secondary science education. | The SRL-aware chatbot supports stronger self-regulation strategies, motivation, and personalized feedback than a rule-based system. |
| *Student–AI Interaction: A Case Study of CS1 Students* | Amoozadeh, M.; Nam, D.; Prol, D.; Alfageeh, A.; Prather, J.; Hilton, M.; Srinivasa Ragavan, S.; Alipour, A. | Mixed-methods case study with 15 CS1 students using ChatGPT integrated into the programming environment. | Shows frequent reliance on GenAI, limited verification behaviors, and mixed effects on self-efficacy; highlights risks of overreliance and need for SRL scaffolding. |
| *Modeling AI-Assisted Writing: How Self-Regulated Learning Influences Writing Outcomes* | Jin, F.; Lin, C.-H.; Lai, C. | Large-scale survey study (n ≈ 1073 postgraduates, 21 countries) analyzed using structural equation modeling. | Demonstrates that SRL predicts productive AI use; advanced transformative use relates to better writing quality, motivation, and critical thinking. |
| *Cognitive and Sociocultural Dynamics of Self-Regulated Use of Machine Translation and Generative AI Tools in Academic EFL Writing* | Wang, Y. | Mixed-methods study with 79 EFL learners combining surveys and qualitative reflections. | Learners perceive improved writing support from AI, but instructor guidance and peer interaction remain essential and irreplaceable. |
| *The Mediating Role of Generative AI Self-Regulation on Students’ Critical Thinking and Problem-Solving* | Zhou, X.; Teng, D.; Al-Samarraie, H. | Quantitative survey study (n = 223) analyzed via structural equation modeling. | Finds that self-regulation mediates the relationship between ease of use and critical thinking/problem-solving; usefulness and learning value show weaker effects. |
| *Taking Responsibility for Meaning and Mattering: An Agential Realist Approach to Generative AI and Literacy* | Kumar, P. C.; Cotter, K.; Cabrera, L. Y. | Conceptual / theoretical article grounded in agential realism and literacy theory. | Reframes GenAI concerns from authorship and authenticity toward responsibility and ethical co-construction of meaning in human–AI literacy practices. |
| *Beyond the Deepfake Problem: Benefits, Risks and Regulation of Generative AI Screen Technologies* | Broinowski, A.; Martin, F. R. | Conceptual policy and media analysis (abstract-level). | Argues that governance must move beyond narrow deepfake narratives toward broader regulation of generative media technologies. |
| *Ethical Dimensions of Generative AI: A Cross-Domain Analysis Using Machine Learning Structural Topic Modeling* | Ali, H.; Aysan, A. F. | Computational literature analysis using Structural Topic Modeling on 364 Scopus documents (2022–2024). | Identifies dominant ethical themes across domains, supporting the need for domain-specific ethical guidelines for GenAI. |
| *ChatGPT and Dental Education: Opportunities and Challenges* | Elnagar, M. H.; Yadav, S.; Venugopalan, S. R.; Lee, M. K.; Oubaidin, M.; Rampa, S.; Allareddy, V. | Narrative review / conceptual analysis focused on dental education. | Highlights risks such as plagiarism, misinformation, and bias, and calls for domain-specific guidelines and validation practices. |
| *Education and Training Assessment and Artificial Intelligence: A Pragmatic Guide for Educators* | Newton, P. M.; Jones, S. | Practical guide and conceptual discussion on assessment validity in the AI era. | Advocates shifting assessment toward authenticity, process evidence, and competence-based evaluation to preserve validity. |
| *Artificial Intelligence and Public Health: Prospects, Hype and Challenges* | Nutbeam, D.; Milat, A. J. | Conceptual and policy-oriented analysis synthesizing public health and AI literature. | Warns against AI hype; emphasizes governance, validation, ethical oversight, and evidence-based adoption. |
| *Reimagining Higher Education: Navigating the Challenges of Generative AI Adoption* | Hughes, L.; Malik, T.; Dettmer, S.; Al-Busaidi, A. S.; Dwivedi, Y. K. | Conceptual synthesis and agenda-setting paper on institutional adoption readiness. | Identifies policy, assessment, faculty development, and equity challenges; recommends coordinated institutional strategies. |
| *Perception of the Risks Inherent in New AI Technologies* | Machleidt, P.; Mráčková, J.; Mráček, K. | Conceptual analysis grounded in technology assessment and precautionary principles. | Argues for balanced AI governance combining regulation, education, and innovation-friendly policies. |
| *Friend or Foe? Exploring the Implications of Large Language Models on the Science System* | Fecher, B.; Hebing, M.; Laufer, M.; Pohle, J.; Sofsky, F. | Conceptual analysis of LLM impacts on research and science systems. | Discusses systemic risks and governance needs for research ecosystems under widespread LLM adoption. |
| *The Promise and Challenges of Generative AI in Education* | Giannakos, M.; Azevedo, R.; Brusilovsky, P.; Cukurova, M.; Dimitriadis, Y.; Hernandez-Leo, D.; Järvelä, S.; Mavrikis, M.; Rienties, B. | Commentary / synthesis paper (abstract-level). | Highlights pedagogical opportunities alongside governance, ethics, and SRL challenges in GenAI-enabled education. |
| **Full title** | **Authors** | **Methods (from accessible abstract/text)** | **Key results (from accessible abstract/text)** |
| *A Self-Regulated Learning Framework Using Generative AI and Its Application in CS Educational Intervention Design* | Prasad, P.; Sane, A. | Conceptual paper proposing a self-regulated learning (SRL) framework for novice programmers using generative AI; focuses on intervention design rather than empirical testing. | Conceptualizes GenAI as interacting with learners’ self-regulation processes; outlines an SRL-guided design space for educational interventions that emphasize planning, monitoring, and reflection rather than answer provision. |
| *Attaining Self-Regulation: A Social Cognitive Perspective* | Zimmerman, B. J. | Theoretical chapter (non-empirical) presenting a social-cognitive model of self-regulated learning. | Provides the foundational SRL model (goal setting, self-monitoring, self-evaluation) widely used to frame learning designs and metacognitive scaffolding. |
| *A Pedagogical Design for Self-Regulated Learning in Academic Writing Using Text-Based Generative AI Tools: 6-P Pedagogy* | Kong, S.-C.; Lee, J. C.-K.; Tsang, O. | Conceptual pedagogical design paper proposing a six-phase framework (Plan–Prompt–Preview–Produce–Peer-review–Portfolio-tracking); explicitly notes lack of empirical validation. | Argues that structured phases, verification, and reflective portfolio work can foster SRL, critical thinking, and academic integrity when using GenAI for writing. |
| *Educational Design Principles of Using AI Chatbot That Supports Self-Regulated Learning* | Chang, D. H.; Lin, M. P.-C.; Hajian, S.; Wang, Q. Q. | Conceptual design-principles paper grounded in SRL and Judgments of Learning theory; no original empirical study reported. | Proposes that chatbots should scaffold goal setting, feedback-based self-assessment, and personalization, positioning AI as a learning resource rather than an answer generator. |
| *Re-Examining the Future Prospects of Artificial Intelligence in Education in Light of the GDPR and ChatGPT* | Bai, J. Y.; Zawacki-Richter, O.; Muskens, W. | Conceptual and governance-oriented analysis revisiting earlier AI-in-education scenarios in light of GDPR and ChatGPT. | Highlights regulatory, assessment, and governance implications of generative AI; emphasizes participatory approaches and student agency in institutional AI policies. |
| *Designing Embodied Generative Artificial Intelligence in Mixed Reality for Active Learning in Higher Education* | Nguyen, A.; Gul, F.; Dang, B.; Huynh, L.; Tuunanen, T. | Design Science Research study developing an embodied GenAI system in mixed reality; evaluated with 26 higher-education learners. | Reports increased learner engagement and active learning; examines how embodied GenAI can support learning while managing cognitive load in immersive environments. |
| *Generative AI and Learning Analytics: Pushing Boundaries, Preserving Principles* | Khosravi, H.; Shibani, A.; Jovanovic, J.; Pardos, Z. A.; Yan, L. | Editorial and conceptual synthesis framing a special issue; uses learning analytics theory (e.g., Clow’s cycle) to organize emerging work. | Positions learning analytics as a lens to evaluate GenAI’s impact on learning and agency; argues for human-centered, transparent, and privacy-preserving integration of LA and GenAI. |
| *AI and English Language Teaching: Affordances and Challenges* | Crompton, H.; Edmett, A.; Ichaporia, N.; Burke, D. | Systematic review (PRISMA-guided) synthesizing 42 studies on AI in English language teaching (based on abstract). | Identifies affordances such as personalization and feedback alongside challenges including reliability, bias, and over-standardization; calls for nuanced and context-aware adoption. |
| *Microlearning and Generative AI for Pre-Service Teacher Education* | Kohnke, L.; Zou, D.; Xie, H. | Qualitative case study over 13 weeks with 19 pre-service teachers using microlearning modules with GenAI. | Reports progressive development of teachers’ AI integration capability, emphasizing hands-on microlearning to support TPACK and SRL. |
